# Secondary transmission of COVID-19 in preschool and school settings in northern Italy after their reopening in September 2020: a population-based study

**DOI:** 10.2807/1560-7917.ES.2020.25.49.2001911

**Published:** 2020-12-10

**Authors:** Elisabetta Larosa, Olivera Djuric, Mariateresa Cassinadri, Silvia Cilloni, Eufemia Bisaccia, Massimo Vicentini, Francesco Venturelli, Paolo Giorgi Rossi, Patrizio Pezzotti, Emanuela Bedeschi, the Reggio Emilia Covid-19 Working Group, Reggio Emilia Covid-19 Working Group, Reggio Emilia Covid-19 Working Group, Massimo Costantini, Paolo Giorgi Rossi, Roberto Grilli, Massimiliano Marino, Debora Formisano, Giulio Formoso, Emanuela Bedeschi, Cinzia Perilli, Ivano Venturi, Eufemia Bisaccia, Elisabetta Larosa, Mariateresa Cassinadri, Silvia Cilloni, Cinzia Campari, Francesco Gioia, Serena Broccoli, Marta Ottone, Pierpaolo Pattacini, Giulia Besutti, Valentina Iotti, Lucia Spaggiari, Pamela Mancuso, Andrea Nitrosi, Marco Foracchia, Rossana Colla, Alessandro Zerbini, Marco Massari, Anna Maria Ferrari, Mirco Pinotti, Nicola Facciolongo, Ivana Lattuada, Laura Trabucco, Stefano De Pietri, Giorgio Francesco Danelli, Laura Albertazzi, Enrica Bellesia, Simone Canovi, Mattia Corradini, Tommaso Fasano, Elena Magnani, Annalisa Pilia, Alessandra Polese, Silvia Storchi Incerti, Piera Zaldini, Efrem Bonelli, Bonanno Orsola, Matteo Revelli, Carlo Salvarani, Francesco Venturelli

**Affiliations:** 1 Public Health Unit, Azienda Unità Sanitaria Locale – IRCCS di Reggio Emilia, Reggio Emilia, Italy; 2 Epidemiology Unit, Azienda Unità Sanitaria Locale – IRCCS di Reggio Emilia, Reggio Emilia, Italy; 3 Center for Environmental, Nutritional and Genetic Epidemiology (CREAGEN), Section of Public Health, Department of Biomedical, Metabolic and Neural Sciences, University of Modena and Reggio Emilia, Italy; 4 Clinical and Experimental Medicine PhD Program, University of Modena and Reggio Emilia, Modena, Italy; 5 Department of Infectious Diseases, Istituto Superiore di Sanità, Rome, Italy; 6 The members of the Reggio Emilia Covid-19 Working Group are listed under Investigators

**Keywords:** SARS-CoV-2, Covid-19, Schools, outbreaks, surveillance

## Abstract

We report epidemiological investigations of transmission of the severe acute respiratory syndrome coronavirus 2 (SARS-CoV-2) in 41 classes of 36 schools in Reggio Emilia province, northern Italy, from their reopening on 1 September to 15 October 2020. The overall secondary case attack rate was 3.2%, reaching 6.6% in middle and high schools. More timely isolation and testing of classmates could be effective in reducing virus transmission in this setting.

Schools in Reggio Emilia province, northern Italy, reopened on 1 September 2020 after a long period of closure due to lockdown and summer holidays. We conducted epidemiological investigations after reopening in 41 classes in 36 different educational settings in this province after the notification of an infection with severe acute respiratory syndrome coronavirus 2 (SARS-CoV-2).

## Current epidemiological features of COVID-19 in Reggio Emilia province and reopening of educational facilities

The first wave of the coronavirus disease (COVID-19) pandemic hit the Reggio Emilia province (northern Italy, 530,000 inhabitants) in March and April 2020, reaching ca 0.9% cumulative incidence (0.4% for age < 50 years and 3.2% for age > 80 years) with a more than 15% fatality rate (0.2% for age < 50 years and 33.4% for age > 80 years) [1,2]. After 5 months of low incidence, the province began the second wave in October (Figure).

**Figure:**
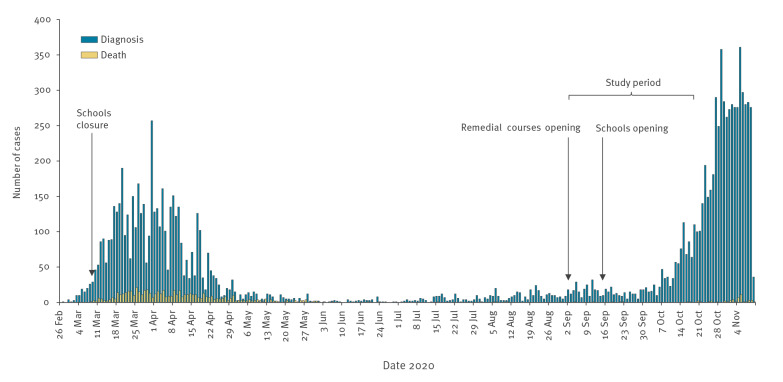
Daily number of notified COVID-19 cases and deaths since the start of the epidemic in Italy, Reggio Emilia province, 27 February–10 November 2020 (n = 11,878 cases, n = 658 deaths)

COVID-19: coronavirus disease.

Ca 31,000 students aged 0–19 years are attending educational institutions in the province: infant-toddler centres (age 0–3 years), preschools (age 3–5 years), elementary schools (age 6–10 years), middle schools (age 11–13 years) and high schools (age 14–19 years). While infant-toddler centres and preschools reopened after lockdown on 1 September and remedial courses were activated, the official reopening of all schools was on 15 September.

## Physical distancing policies in schools

Upon reopening of all schools, the following physical distancing measures were adopted (Supplementary Table S1): mandatory wearing of surgical masks for children at all times except when students are seated at their desk and are not speaking (except in preschools or elementary schools where wearing the mask is never mandatory); only single desks are used (rather than the traditional double desks), and desks must be at least 1 m apart; crowding at separate school entrances and exits is minimised by creating temporal and spatial pathways for the different classes; mixing classes for curricular activities is minimised; all extra-curricular activities have been suspended [3]. In some schools, when the classrooms are not big enough to respect physical distancing, students are divided into two groups, which alternate attending school and remote learning. 

## Study period and epidemiological investigation

We included all consecutive COVID-19 cases, confirmed to be positive with RT-PCR for SARS-COV-2 infection, diagnosed from 1 September to 15 October in Reggio Emilia province among children and adolescents (0–19 years) who had possible exposure or contacts in school assessed during the epidemiological investigation. We excluded cases that occurred among children not attending schools in the period investigated for contact tracing, i.e. in the period starting 48 h before symptom onset and for asymptomatic cases 48 h before diagnosis or 48 h after the contact with a certain case, whichever occurred first (n = 134).

All SARS-CoV-2-positive swabs are immediately reported to the Public Health Department of the Local Health Authority. When a case is identified among students and/or school staff, all classmates and staff who had contact with the index case are immediately tested and retested 14 days after the last contact with the index case if the first test was performed more than 10 days after the contact; usually only one test was performed. During the investigation, the nature of the contact between the index case and their classmates determines isolation measures: (i) all students are isolated if the physical classroom itself makes maintaining distance impossible and/or masks are not worn constantly and/or if secondary cases occur; or (ii) only those in close contact or who have contact outside of school are isolated, provided that physical distancing with the other students has been respected.

### Ethical statement

The study was approved by the Area Vasta Emilia Nord Ethics Committee (no. 2020/0045199). The Ethics Committee authorised to use patients’ data even in the absence of consent if it would not be possible to contact them, given that all the efforts to contact them have done; none denied consent.

## Secondary attack rate

In the study period, 41 classes in 36 schools were notified: eight infant-toddler centres and preschools, 10 elementary and 18 secondary schools (middle and high schools), including a total population of 1,248 individuals (209 teachers/staff and 1,039 children) (Table 1). During the epidemiological investigations, 1,200 contacts were identified, of whom 1,198 were tested (994 students and 204 teachers/staff). 

**Table 1:** Characteristics of teaching/childcare facilities with identified COVID-19 cases and their pupils and personnel, Reggio Emilia province, Italy, 1 September–15 October 2020 (n = 1,248)

	Number	Percentage
Number of schools	36	
Type of school		
Infant-toddler centre and preschool (0–5 years)	8	
Elementary school (6–10 years)	10	
Secondary school	18	
Middle school (11–13 years)	5	
High school (14–19 years)	13	
Number of school classes	41	
Index cases	48	
Students	43	
Teachers/staff	5	
Number of contacts identified during investigations	1,200	100
Tested students	994	82.8
Students not tested	2	0.2
Tested teachers/staff	204	17.0
Secondary cases	38	3.2^a^
Students	38	
Teachers/staff	0	

COVID-19: coronavirus disease.

^a ^The denominator here was the tested contacts, i.e. 1,198.

A total of 38 secondary cases (3.8%) were identified among the 994 tested children: in one of 10 elementary schools and in two of five middle schools and six of 13 high schools (Table 2). There was no secondary transmission among teachers resulting in an overall attack rate of 3.2%. The attack rate was higher in secondary schools (middle and high schools) (6.6%) than in elementary schools (0.38%), while there were no secondary cases in the preschools or among teachers/staff. The mean age of the index cases was 13.3 years (range: 10–17 years); of the positive contacts it was 13.2 years (range: 10–18 years).

**Table 2:** Secondary attack rates for COVID-19, by level of educational facility, Reggio Emilia province, Italy, 1 September–15 October 2020 (n = 1,198)

Type of educational setting	Number of index cases	Number of secondary cases	Number of contacts	Attack rate
Infant-toddler centre/preschool	6 children and 2 teachers	0	156	0
Elementary school	14 children	1	266	0.38%
Secondary school (middle and high schools)	23 children and 5 teachers/staff	37	572	6.46%
Total students	43	38	994	3.82%
Teachers/staff	5	0	199	0
Overall (students and staff)	48	38	1,198	3.17%

COVID-19: coronavirus disease.

## Description of school clusters

Table 3 summarises the information about each of the nine clusters. The index case in the elementary school classroom (Cluster 1) had most probably been infected by a family member outside the household. All the classmates and teachers were tested and only one asymptomatic secondary case was found.

**Table 3:** Characteristics of primary and secondary COVID-19 cases and potential sources of infection^a^, Reggio Emilia province, Italy, 1 September–15 October 2020 (n = 50)

Type of school	Cluster	Possible source of infection	Symptoms onset of the index case	Days from symptom onset to swab positivity of the index case	Number of secondary cases	Last contact with index case^b^	Symptom onset of the secondary case^c^	Swab positivity of secondary case^d^	Previous other contact with household/family members who were positive to SARS-CoV-2
Elementary	1	Household/family contact member outside the household positive for SARS-CoV-2	Symptoms Day 0	0	1	Day +1	Asymptomatic	Day +4	No
High school	2	Household contact who was in isolation following positive swab for travel screening	Asymptomatic	0	3	Day −1	Day +11	Day +14	No
						No info	Day +7	Day +14	No
						No info	Day +10	Day +17	No
High school	3	Isolated since Day −5 due to positivity of household member who had been symptomatic since Day −13	Asymptomatic	0	7	Day −5	Day −8	Day −2	No
							Day −8	Day +2	No
							Asymptomatic	Day +2	No
							Asymptomatic	Day +2	No
							Day −8	Day +2	No
							Asymptomatic	Day +7	No
							Asymptomatic	Day +3	No
High school	4	No previous contacts reported	Symptoms Day 0	10	1	No info	Day +4	Day +8	No
			Asymptomatic	0					
High school	5	Day −2: last contact with SARS-CoV-2-positive household contact	Asymptomatic	0	1	No info	Asymptomatic	Day +5	No
			Asymptomatic	1					
High school	6	Day +3: contact of a SARS-CoV-2-positive person	Symptoms Day 0	7	1	Day +2	Day +4	Day +7	Household member also symptomatic
High school	7	Day -2: last contact with SARS-CoV-2-positive household member	Asymptomatic	0	1	No info	Asymptomatic	Day +2	No
Middle school	8	No previous contacts reported	Symptoms Day 0	9	2	No info	Asymptomatic	Day +11	No
						Day +8	Asymptomatic	Day +20	No
Middle school	9 (Teacher 1)	Day -6: contact of a SARS-CoV-2-positive person	Symptoms Day 0	3	21	Day 0	Day +6	Day +8	No
							Asymptomatic	Day +19	No
							Asymptomatic	Day +19	No
							Asymptomatic	Day +19	No
							Asymptomatic	Day +20	No
	9 (Teacher 2)	No previous contacts reported	Symptoms Day 0	6		Day 0	Day +3	Day +7	Day +2: possible contact in a leisure activity; day +3: possible contact in another leisure activity where cases were reported.
							Asymptomatic	Day +14	No
							Day +8	Day +14	No
							Day +9	Day +14	No
	9 (Teacher 2)	See above				Day +1	Day +9	Day +10	Day +8: leisure activity
							Day +5	Day +14	No
							Day +9	Day +21	No
							Asymptomatic	Day +16	No
							Asymptomatic	Day +16	No
							Asymptomatic	Day +16	Day +15: contact with positive person
	9 (Teachers 1 and 2)	See above				Day 0	Day +6	Day +17	No
							Asymptomatic	Day +16	No
							Asymptomatic	Day +16	No
							Day +6	Day +16	No
	9 (Teacher 2)	See above				Day +1	Asymptomatic	Day +19	No
							No info	Day +19	No

COVID-19: coronavirus disease; Day 0: disease onset of the index case (either date of symptom onset or date of positive swab); SARS-CoV-2: severe acute respiratory syndrome coronavirus 2.

^a^ Policies described (Supplementary Table S1) were in place in all the schools during the period of the clusters.

^b^ Days from the start of the symptoms or positive swab of the index case until the last contact of the secondary case with index case.

^c ^Days since symptoms onset of the index cases until symptom onset of the secondary case.

^d^ Days since symptoms onset or swab positivity of the index case until the swab positivity of the secondary case.

Cluster 2 was identified when a student tested positive for SARS-CoV-2 after a household member was found positive who had travelled back form a high-incidence area. All classmates and teachers were tested, with three positive cases identified, all of whom developed mild symptoms. No other possible sources of transmission were identified for the secondary cases.

After the almost simultaneous reporting of two cases in one class, an investigation started in another high school (Cluster 3): one symptomatic subject tested positive in mid-October and one contact of a family cluster tested positive the day after. All classmates and teachers were tested and isolated. Six resulted positive, two of whom reported mild symptoms 8 days earlier at the beginning of October. Analysis of the possible infection sources outside of school and dates of symptoms made it possible to identify the asymptomatic positive case in mid-October as the only index case.

Investigations of Clusters 5 and 7 in two high schools started after three asymptomatic household members reported contact with a symptomatic household member. Each cluster had one secondary case. In Cluster 6, both the index case and the secondary case had previous contact with a positive person, and the temporal association was difficult to establish. 

For Clusters 4 and 8, no possible sources of infection were identified. No other possible sources of transmission were identified for the secondary cases, although one of the secondary cases reported out-of-school contact with the index case.

Cluster 9 involved five classes in three high schools under the same administration Teachers 1 and 2 were each active in more than one school. The index cases were most probably two teachers active in all three schools. Only three secondary cases in two classes reported a possible contact outside of school, but in all cases, analysis of the date of symptom onset made the alternative route of infection unlikely. For the other three classes, no other contacts outside of school were identified.

## Discussion

Secondary cases occurred in nine clusters and generated 38 secondary cases, with an attack rate of 3.8%. Previous studies measuring incidence in school-aged children and adolescents before and during school closures suggested limited transmission in schools [4-6]. Contact-tracing studies conducted in schools and educational settings in Australia, Singapore and Ireland found a low rate of, or even no, secondary cases [7-9]. One study reporting results of screening at the reopening of kindergartens in Korea found only one possible secondary case among 45 cases identified when attending the school [10]. Similarly, a low transmission from student to student was found in the United Kingdom when analysing predominantly primary and preschools [11] and in Germany for all ages [12]. These findings are in line with our report in terms of transmission in preschools and elementary schools, but not with our results for secondary schools. The policy of not isolating all classmates immediately and delays in testing might explain the difference between the results observed in Germany and ours. On the other hand, one large cluster with a high attack rate among students and teachers has been reported in a high school in Israel [13].

The inclusion criteria (all consecutive cases attending school), the uniform investigation protocol (testing all classmates) and the population-based nature of the study allowed us to estimate an unbiased risk of secondary cases. This report is limited by the small number of clusters but has the advantage of an accurate analysis of the chain of transmission, making it possible to reasonably rule out other sources of infection for secondary cases. Another limit of our investigations is that they could not distinguish between classroom transmissions and those linked to activities and behaviours outside of school, such as using public transportation or leisure activities. Furthermore, it was impossible in two cases to assess exactly for how many days the students shared the same classroom while the index case was still infectious because that case was asymptomatic.

## Conclusions

Transmission within the schools of Reggio Emilia province, northern Italy, occurred in a non-negligible number of cases, particularly in the age group 10–18 years, i.e. in middle and high schools, while no secondary cases were detected in pre-school children, only one case in primary school and no secondary cases among teachers and staff. At least in the largest cluster that we reported, more prompt isolation and testing of classmates could have reduced virus transmission, suggesting the importance of timeliness in this setting.

## Supplementary Data

411000 Supplement
