# Supplementary material for: Secondary transmission of COVID-19 in preschool and school settings in northern Italy after their reopening in September 2020: a population-based study
Source: Euro Surveill. 2020 Dec 10;25(49):2001911. doi: 10.2807/1560-7917.ES.2020.25.49.2001911 (PMC7730487; doi:10.2807/1560-7917.ES.2020.25.49.2001911)
Supplement: Supplement [file 20-01911_DJURIC_Supplement.pdf]

SUPPLEMENTARY MATERIAL

This supplementary material is hosted by Eurosurveillance as supporting information alongside the article “Secondary transmission of COVID-19 in preschool and school settings in northern Italy after their reopening in September 2020: a population-based study”, on behalf of the authors, who remain responsible for the accuracy and appropriateness of the content. The same standards for ethics, copyright, attributions and permissions as for the article apply. Supplements are not edited by Eurosurveillance and the journal is not responsible for the maintenance of any links or email addresses provided therein

Supplementary Table S1. Structural and social distancing measures for the prevention of SARS-CoV-2 transmission in early childhood education facilities and schools in Reggio Emilia, Italy, September–October 2020

Infection control measure	Infant-toddler centres and preschools	Elementary school	Middle school	High school
Wearing surgical masks in the classroom (except when students are seated at their desk and are not speaking)	No	No	Yes	Yes
Wearing surgical masks outside the classroom	No	Yes	Yes	Yes
Single desks	No	Yes	Yes	Yes
Physical distancing between children	No	yes	Yes	Yes
Separate school entrances and exits	No	Yes	Yes	Yes
Suspending extracurricular activities	NA	Yes	Yes	Yes
Ventilation of rooms	Yes	Yes	Yes	Yes
Dividing classes into two groups	No	Some	Some	Some

NA, not applicable
